# Supplementary material for: Tunable Tamm plasmon cavity as a scalable biosensing platform for surface enhanced resonance Raman spectroscopy
Source: Nat Commun. 2023 Nov 4;14:7085. doi: 10.1038/s41467-023-42854-7 (PMC10625559; doi:10.1038/s41467-023-42854-7)
Supplement: Supplementary file 1 — Supplementary Information [file 41467_2023_42854_MOESM1_ESM.pdf]

Supplementary Information for

Tunable Tamm plasmon cavity as a scalable biosensing platform for surface enhanced resonance Raman spectroscopy

Kandammathe Valiyaveedu Sreekanth[1*], Jayakumar Perumal[1,4], U. S. Dinish[1,4], Patinharekandy Prabhathan[2, 3], Yuanda Liu[1], Ranjan Singh[2,3*], Malini Olivo[1,4*] and Jinghua Teng[1*]

[1]Institute of Materials Research and Engineering (IMRE), Agency for Science, Technology and Research (A*STAR), 2 Fusionopolis Way, Innovis #08-03, Singapore, 138634 Republic of Singapore

[2]Division of Physics and Applied Physics, School of Physical and Mathematical Sciences, Nanyang Technological University, 21 Nanyang Link, Singapore 637371, Republic of Singapore

[3]Centre for Disruptive Photonic Technologies, The Photonic Institute, 50 Nanyang Avenue, Singapore 639798, Republic of Singapore

[4]Present address: A*STAR Skin Research Labs (A*SRL), Agency for Science, Technology and Research (A*STAR), 31 Biopolis Way, Nanos #07-01, Singapore, 138669 Republic of Singapore

*Correspondence authors: K. V. S. (sreekanth@imre.a-star.edu.sg), R. S. (ranjans@ntu.edu.sg), M. O. (malini_olivo@asrl.a-star.edu.sg) and J. T. (jh-teng@imre.a-star.edu.sg)

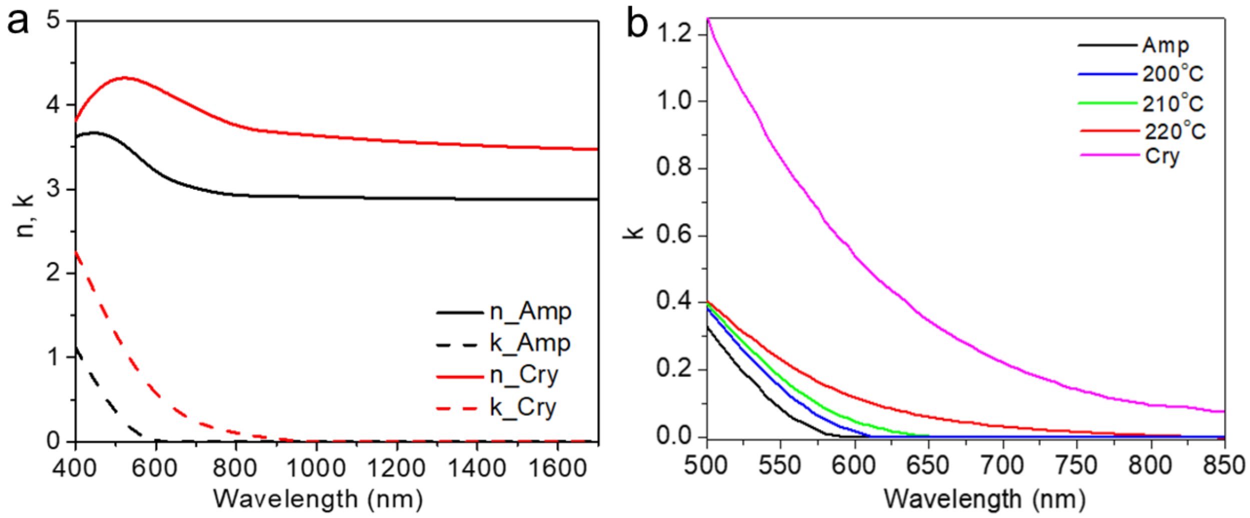

Supplementary Fig. 1. Optical constants of Sb_2S_3. (**a**) Measured optical constants (*n* & *k*) of Sb_2S_3 thin film in amorphous and crystalline phases. (**b**) Measured *k* values of Sb_2S_3 thin film at different annealing temperatures.

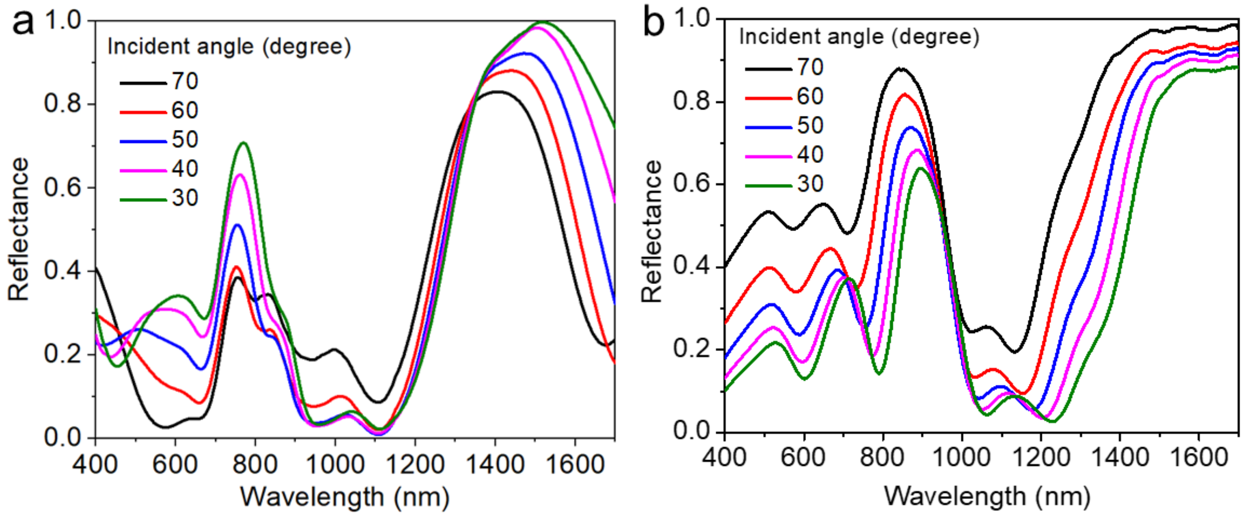

Supplementary Fig. 2. Polarization-dependent angular reflection. Measured angular reflection spectrum of crystalline DBR for (**a**) *p*-polarization and (**b**) *s*-polarization.

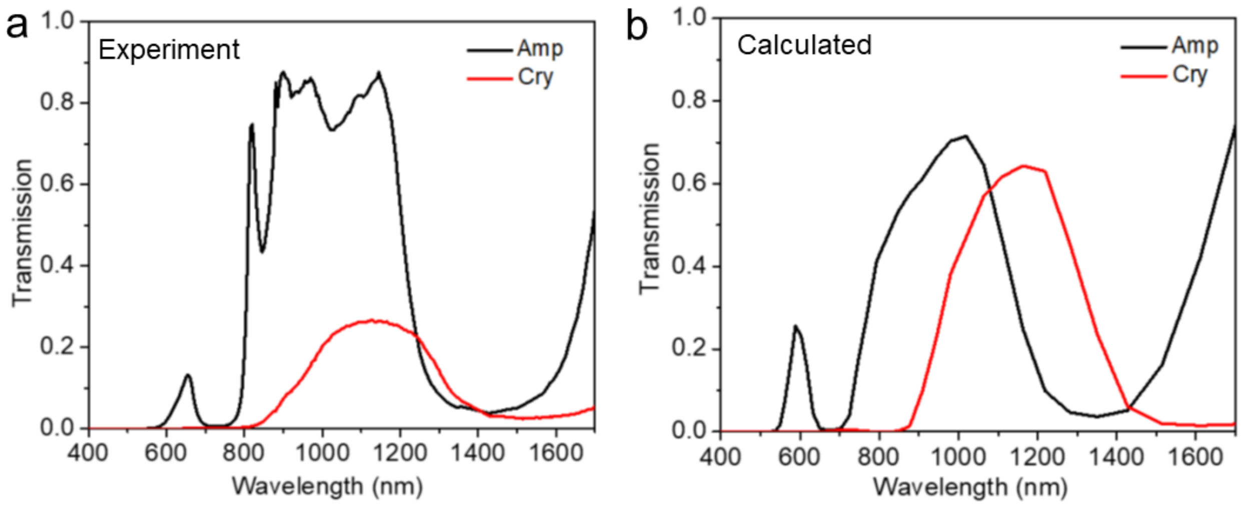

Supplementary Fig. 3. Transmission spectrum of DBR for both phases of Sb_2S_3 at normal incidence (**a**) Measured and (**b**) Calculated.

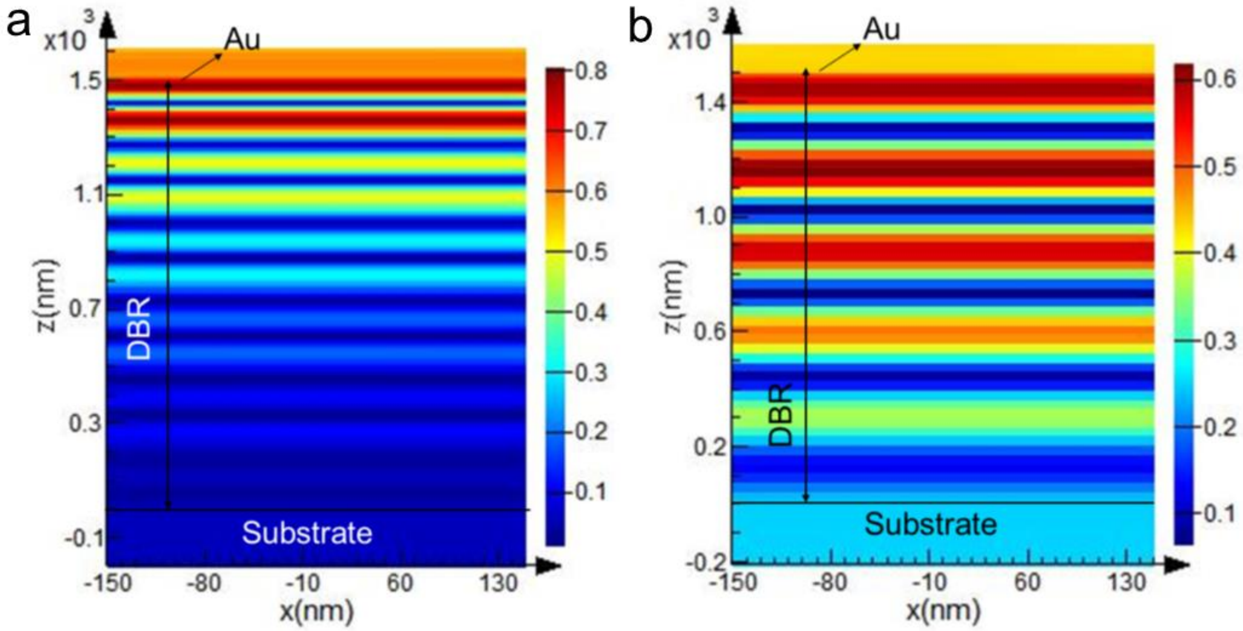

Supplementary Fig. 4. Simulated electric field intensity distribution along the TPP cavity at normal incidence. For the resonance wavelength of (**a**) TPP 2 and (**b**) TPP 1.

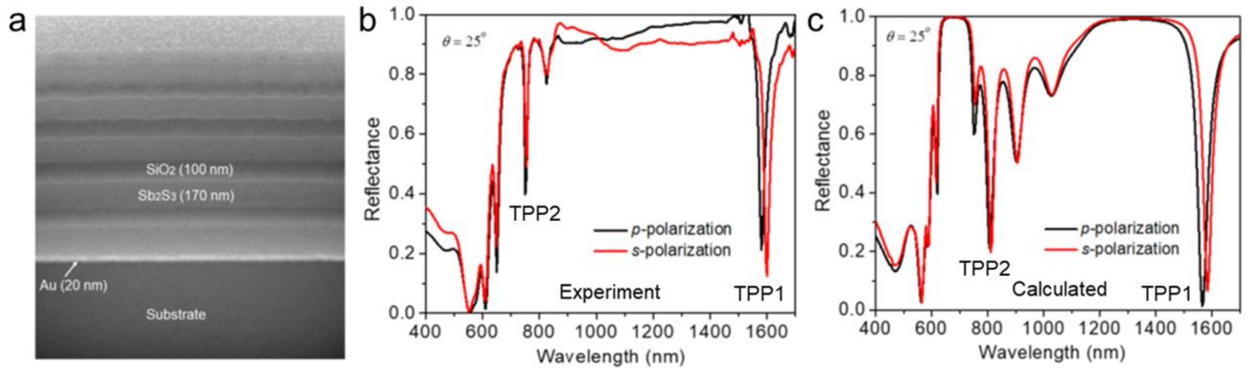

Supplementary Fig. 5. Characterization of TPP cavity with bottom metal layer. (**a**) SEM image of fabricated DBR-Au-substrate TPP cavity. Reflection spectrum for *p*-and *s*-polarizations at an incident angle of 25º (**b**) Measured and (**c**) Calculated.

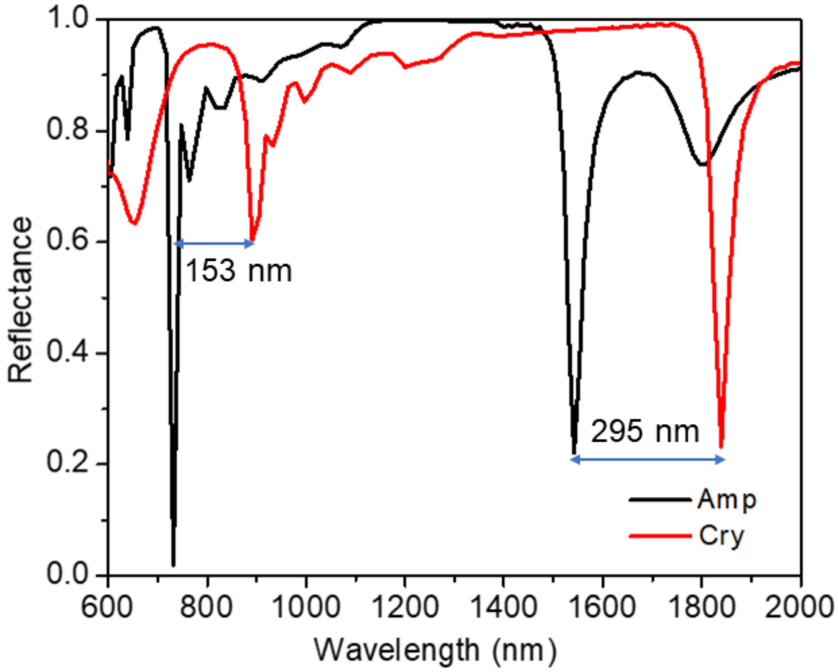

Supplementary Fig. 6. Tunable TPP resonance. Calculated reflection spectrum of TPP cavity for both phases of Sb_2S_3.

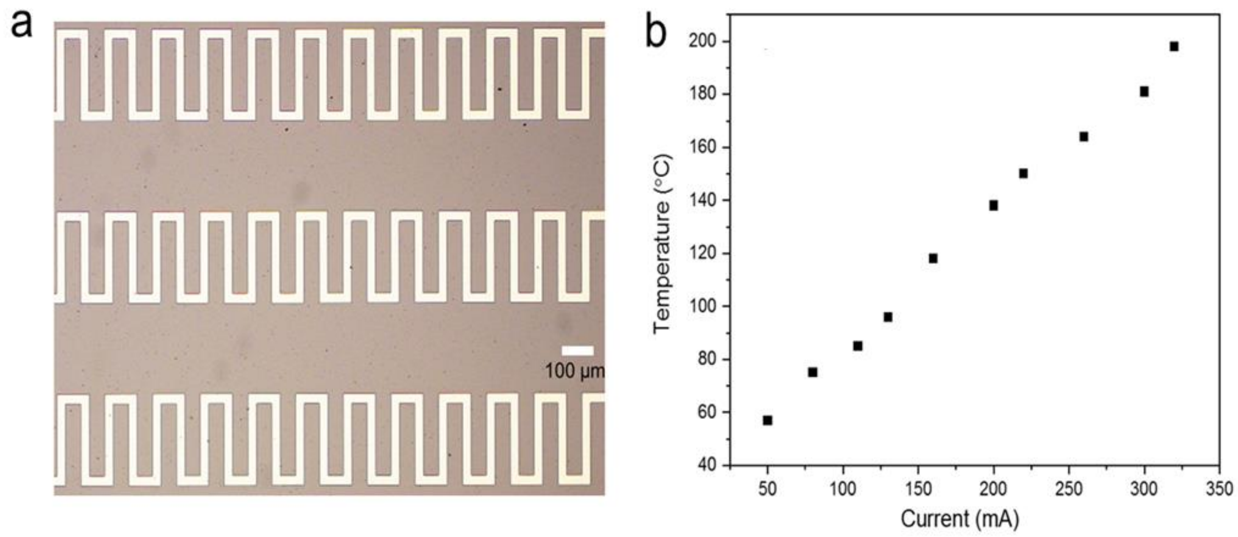

Supplementary Fig. 7. Calibration of microheater. (**a**) Optical microscopy image of the fabricated microheater/Si device and (**b**) temperature calibration of the microheater with applied DC current.

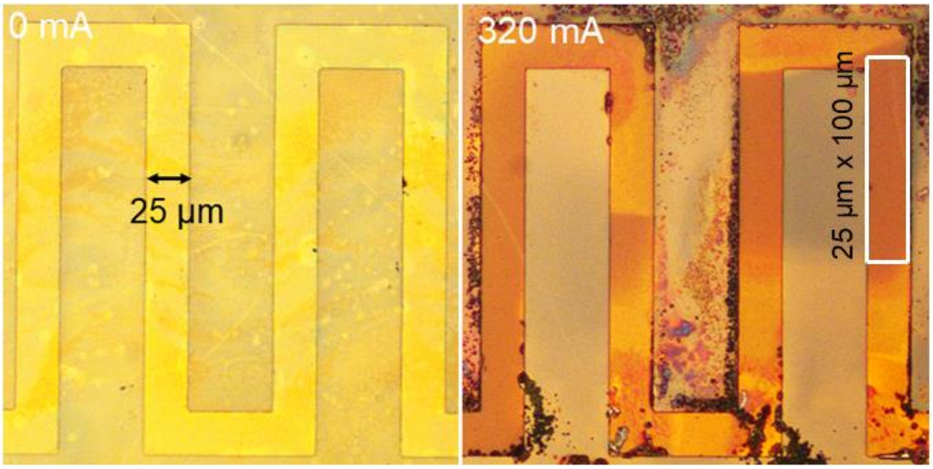

Supplementary Fig. 8. Color change with applied current. Optical microscope image showing the tunable color of Sb_2S_3-SiO_2 DBR with applied DC current.

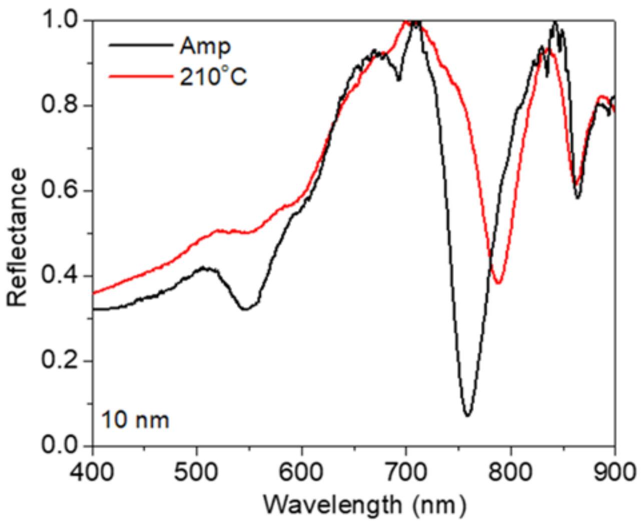

Supplementary Fig. 9. Tuning the TPP resonance. Measured reflection spectrum of 10 nm Au coated TPP cavity (amorphous and annealed at 210°C).

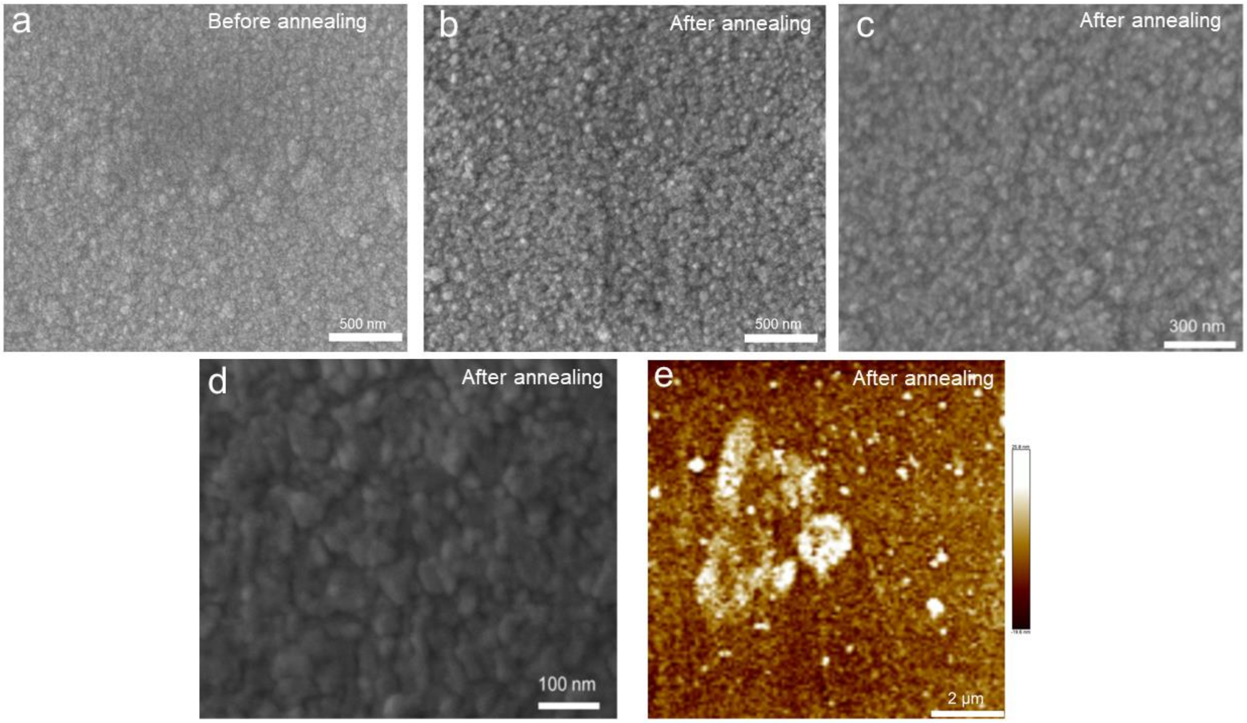

Supplementary Fig. 10. Surface roughness study of TPP cavity. SEM images of the topology of the TPP cavity for different magnifications (**a**) before annealing and (**b**, **c** & **d**) after annealing. (**e**) AFM image of annealed TPP sample (measured surface roughness, R_a = 8.4 nm).

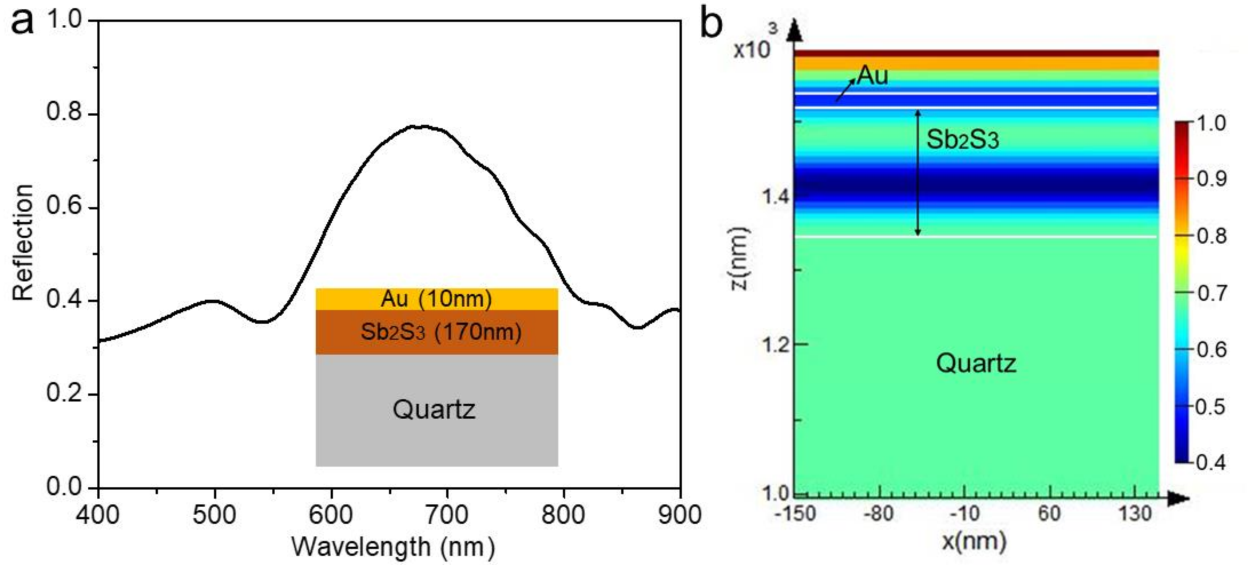

Supplementary Fig. 11. Characterization of reference cavity. (**a**) Measured reflection spectrum of reference cavity at normal incidence. (**b**) Simulated electric field intensity along the stack at 785 nm wavelength and normal incidence.

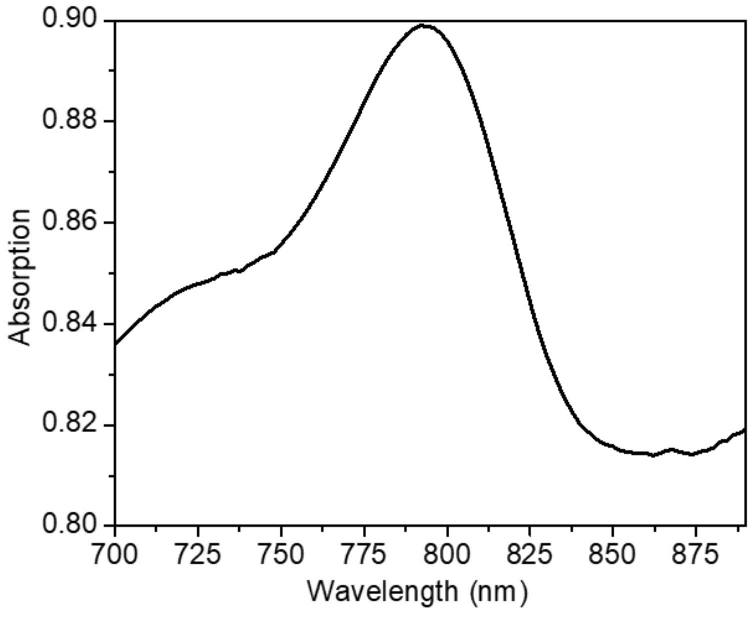

Supplementary Fig. 12. Absorption measurement of dye. Measured absorption spectrum of cyanine 7.5 dye dissolved in DMSO solvent.

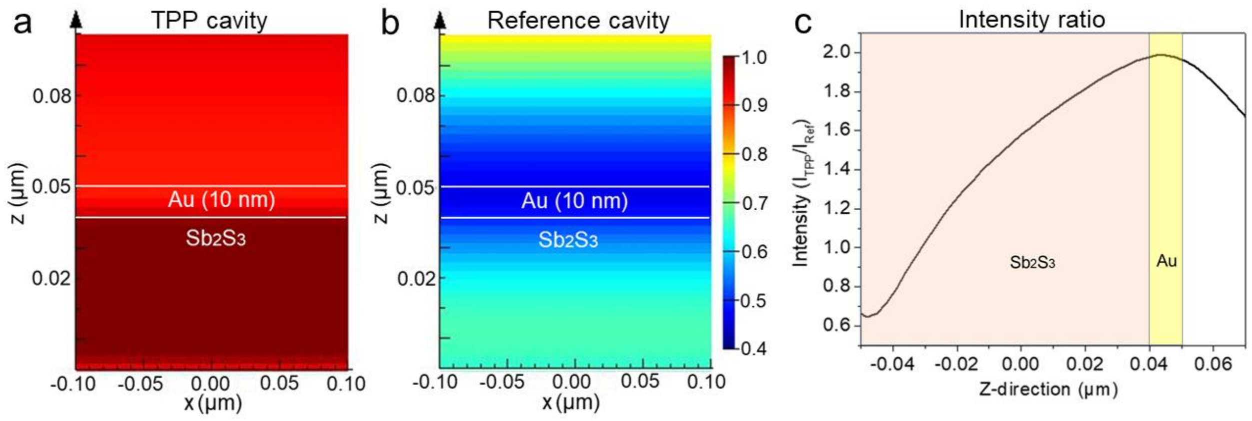

Supplementary Fig. 13. Electric field intensity distribution. 2D cross-sectional map of intensity field distribution at 785 nm, zoomed at Sb_2S_3/Au interface for (**a**) TPP cavity and (**b**) reference cavity. (**c**) The ratio of the intensity field distribution as a function of z-direction.

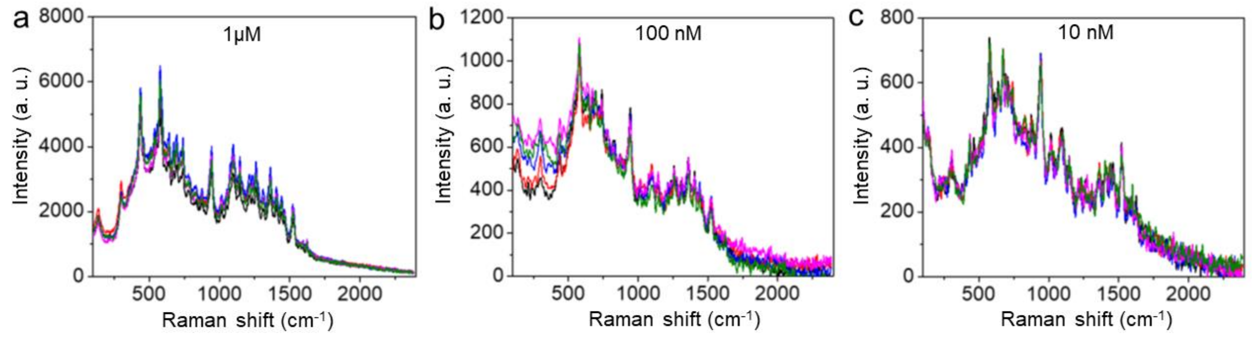

Supplementary Fig. 14. Repeatability study of SERRS. Measured SERRS spectrum of five TPP cavities using cy7.5 dye concentration of (**a**) 1 μM, (**b**) 100 nM, and (**c**) 10 nM.

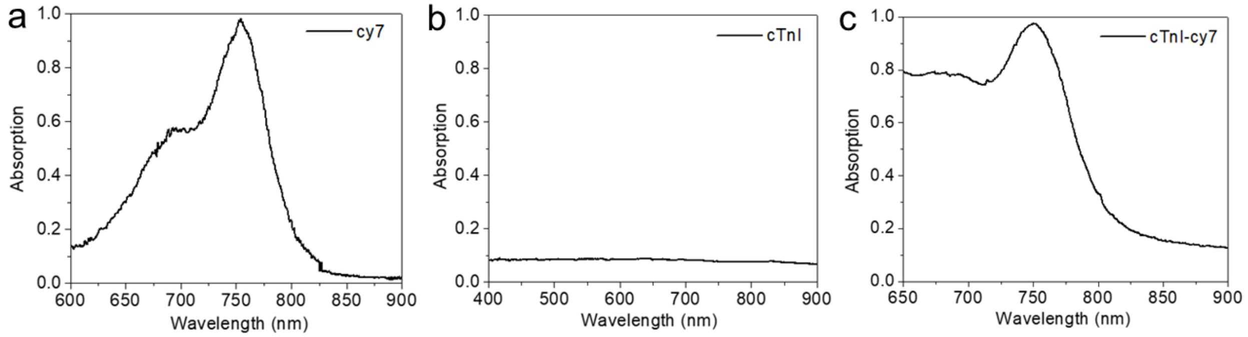

Supplementary Fig. 15. Absorption measurements of dye and protein. Measured absorption spectrum of (**a**) cy 7 dye dissolved in DMSO solvent, (**b**) cTnI alone and (**c**) cy 7 tagged cTnI.

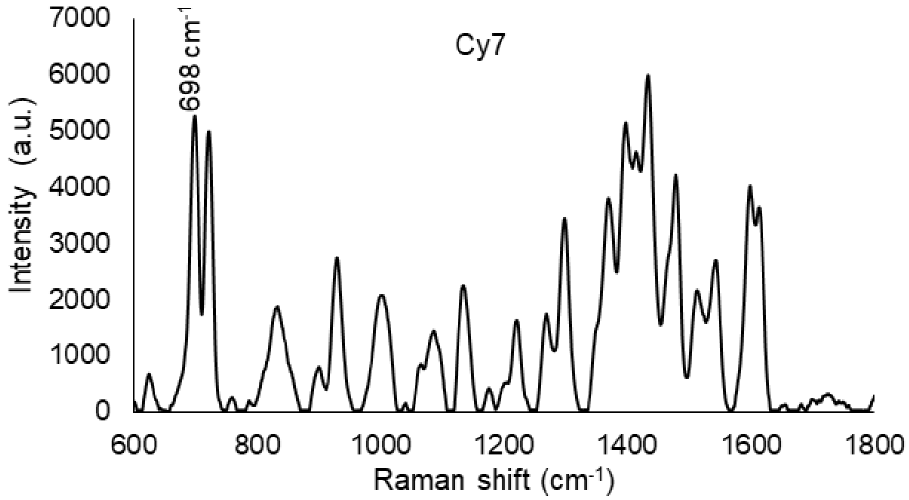

Supplementary Fig. 16. SERS spectrum of cy7 dye. Representative SERS spectrum of cy7 molecule with its prominent peak at 698 cm^{-1} is indicated.

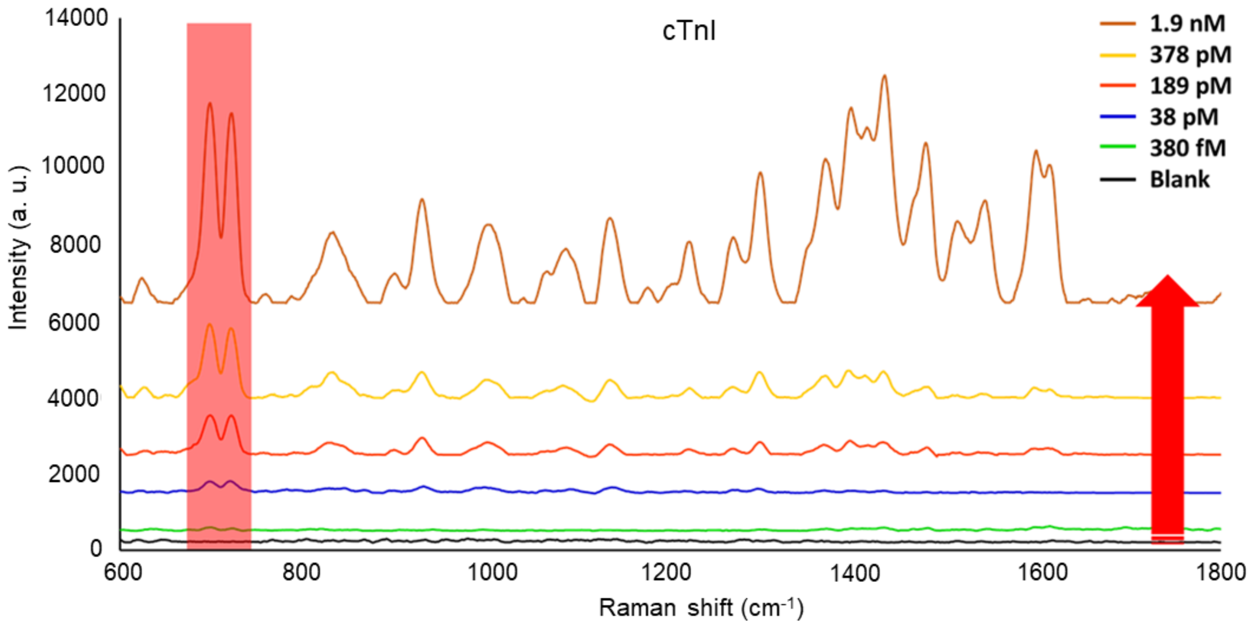

Supplementary Fig. 17. Sensing of different concentrations of cTnI protein. SERRS spectrum of the different concentrations of the cTnI protein with the representative Raman band at 698 cm^{-1} is highlighted in red.

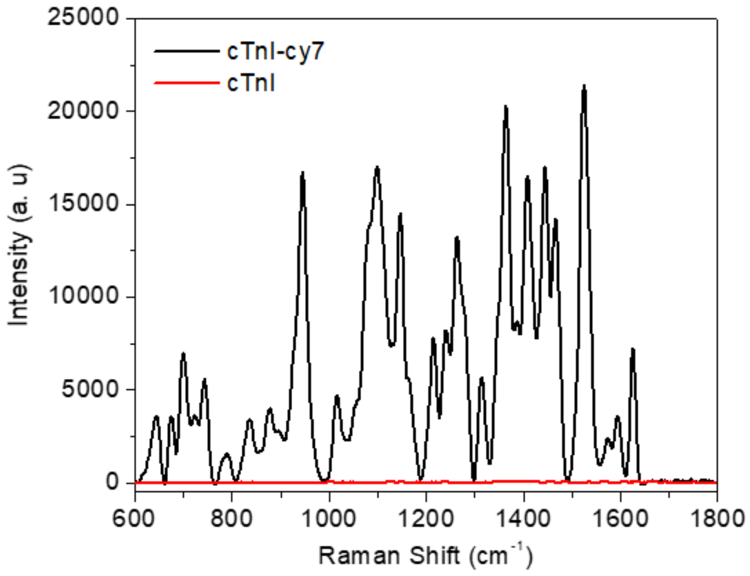

Supplementary Fig. 18. SERS spectrum of cTnI alone and cy7 tagged cTnI.

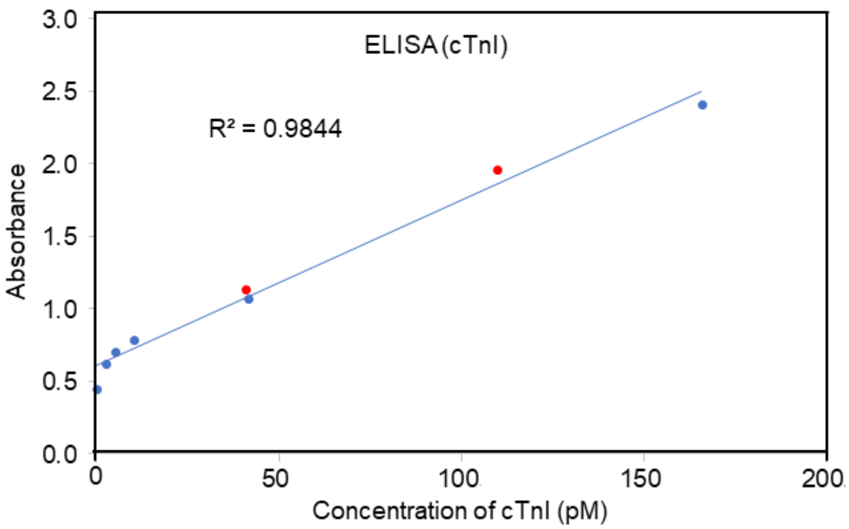

Supplementary Fig. 19. Benchmarking with ELISA test. Calibration plot of ELISA with interpolated spiked cTnI samples (red points).

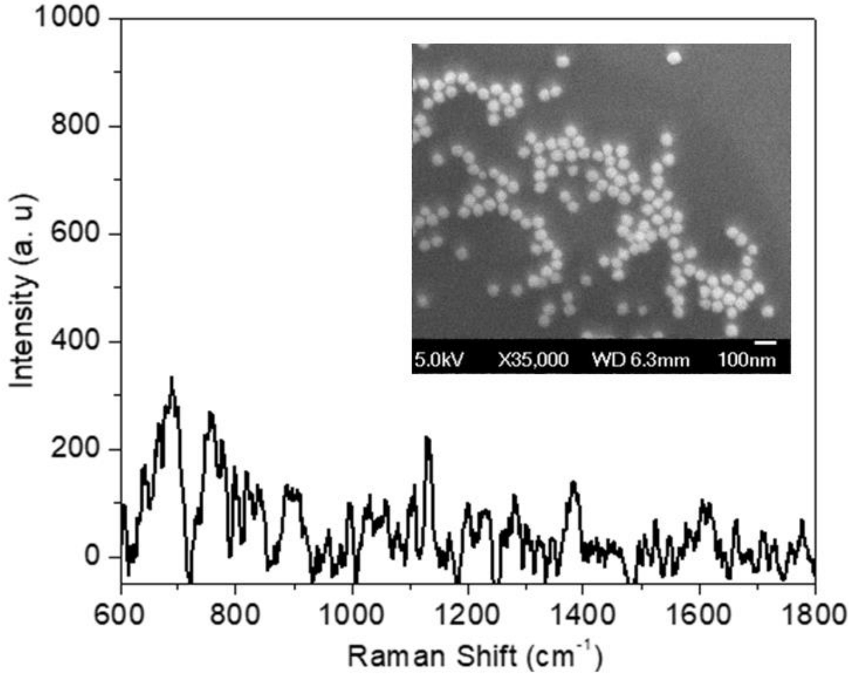

Supplementary Fig. 20. Comparison with Au nanoparticle SERS. SERS spectra of cy7 tagged cTnI protein (38 pM) in Au colloid. A SEM image of ~60 nm diameter Au nanoparticles is shown in the inset.

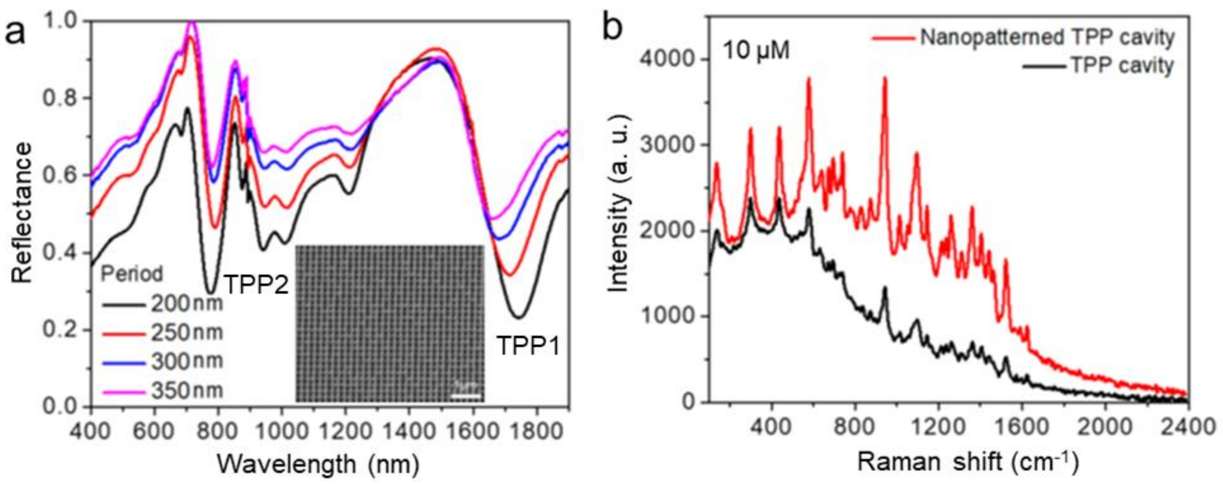

Supplementary Fig. 21. Characterization of nanopatterned TPP cavity. (**a**) Reflectance spectrum of nanopatterned TPP cavity at normal incidence for different grating periods with a hole diameter of 80 nm. The inset shows the SEM image of 2D periodic nanohole grating with a period of 250 nm. (**b**) Measured SERRS spectrum of scalable and nanopatterned (period=250 nm) TPP cavities using 10 µM cy 7.5 concentration. The thickness of Au is 30 nm. All SER(R)S spectra were subtracted from the background (dark) spectra.

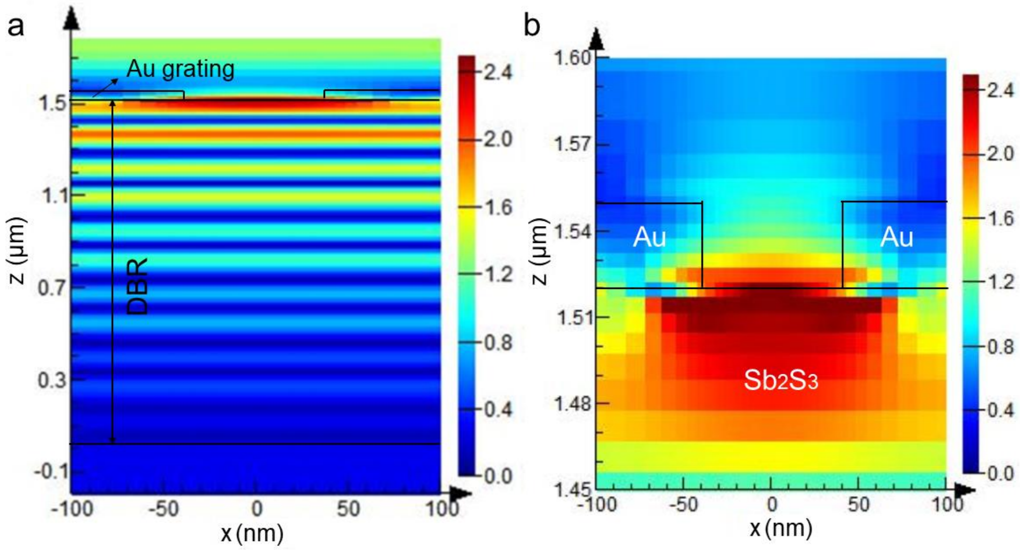

Supplementary Fig. 22. Electric field intensity distribution of nanopatterned TPP cavity (**a**) Simulated electric field intensity distribution along the nanopatterned TPP cavity at normal incidence and TPP 2 resonance wavelength. (**b**) Zoomed image close to the grating region. The field is tightly confined near the nanohole region. Grating parameters: hole diameter=80 nm, grating period=200 nm, and grating depth=30 nm.

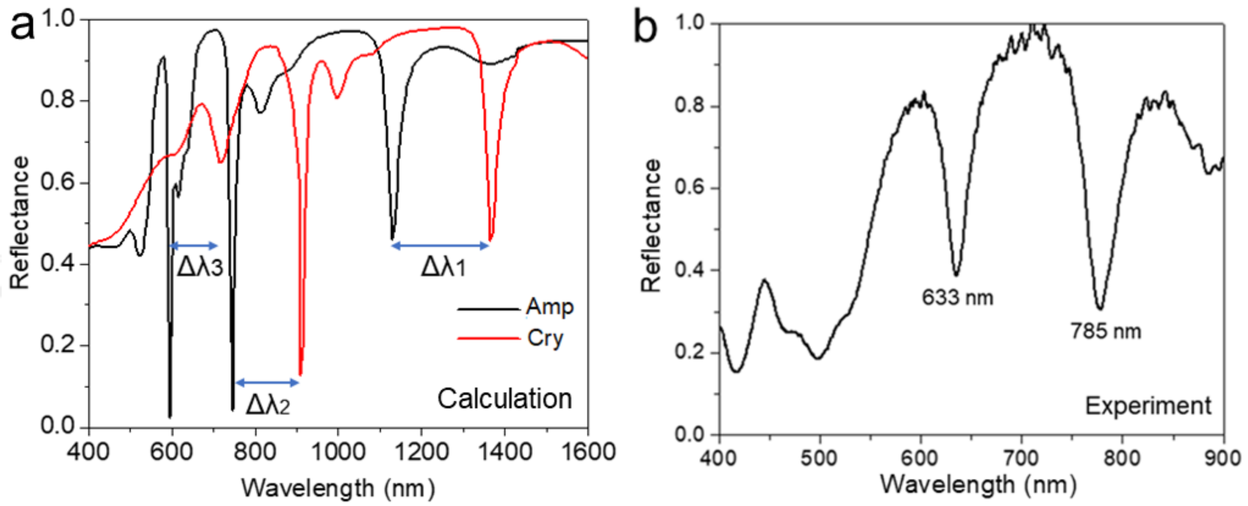

Supplementary Fig. 23. TPP cavity for multiplexed detection. (**a**) Calculated reflection spectrum of TPP cavity with tunable three TPP modes and (**b**) Measured reflection spectrum of TPP cavity (Amp) with two modes.

Supplementary Table 1. Detection of spiked cTnI by ELISA

Actual cTnI concentration	Absorbance	cTnI detected by ELISA
378 pM	1.9525	330 pM
38 pM	1.1272	41 pM
380 fM	0.498	0
190 fM	0.205	0
95 fM	0.403	0
47.5 fM	0.26	0

Supplementary Table 2. Comparison of TPP cavity with existing SERRS demonstrations

Reference	Platform	Scalable	Excitation wavelength (nm)	Plasmonic Resonance matching with absorption peak	Sensitivity
1	Al nanohole array	No	266	Yes	1 nm thick
2	Al nanoparticle array	No	257.2	Yes	Zeptomole
3	Ag nanoparticle	No	514.5	Yes	Attomolar
**Our work**	**TPP cavity**	**Yes**	**785**	**Yes**	**Femtomolar**

Supplementary Note 1. Uniform temperature distribution in the switching area

Here, we demonstrate the uniform temperature distribution on the measured sample area by monitoring the color change of Sb_2S_3-SiO_2 DBR with applied DC current. For this purpose, initially tungsten (200 nm) microheater was fabricated on Si substrates and followed by the deposition of 10 alternating layers of Sb_2S_3 (170 nm) and SiO_2 (100 nm) on the entire area of the Si substrate. Supplementary Fig. 8 shows the optical microscopic image of 'as-deposited' (0 mA) and electrically annealed (320 mA) Sb_2S_3-SiO_2 DBR. It is clear that color change is not uniform throughout the microheater bar due to non-uniform temperature distribution, however, uniform color change is possible for a sample area of 25 μm × 100 μm. The switching area can be further widened by using proper microheater designs.

Supplementary Note 2. Raman mapping of TPP cavity

In order to estimate the uniformity in SERS enhancement from the novel SERRS substrate. We have performed the intensity mapping of a 943 cm^{-1} peak from cy7.5 dye. We were able to clearly observe uniform SERS enhancement from all over the substrate. The high enhancement region is shown in bright red color while relatively low enhancement regions are shown in shades of black, where the dark patches correspond to the area devoid of nanostructures. Certain locations are in shades of black, which indicates defects or irregularity in the structure. The spotted darker points were regions devoid of nanoparticle arrangement in the random island structure. It correlates to the thin film fabrication process. The black mark at the top right corner is due to the small defect in the substrate which is without any random nanoparticles. In general, for SERS substrates, enhancement, and signal reproducibility exhibit inverse correlation. Typically, less than 10-15% variation in intensity between various points of the substrate is well acceptable in SERS measurements, the uniform signal enhancement observed from this substrate is noteworthy. Low signal variation is quite relevant when developing a SERS biosensor where changes in signal intensity are often monitored as a function of analyte concentration.

Supplementary Note 3. Adsorbed molecule sensitivity

To quantify the adsorbed molecules sensitivity, we estimate the sensitivity of the Raman intensity shift to the number of cTnI molecules adsorbed on the sensor surface. For each concentration c of the biomolecule in the sensor, there will be a maximum intensity shift $\Delta I(c)$ with respect to the bare sample. This shift is due to the presence of an average equilibrium population $N(c)$ of adsorbed cTnI molecules on the sensor surface, a number that cannot be directly measured. The sensitivity can be defined as $\Delta I(c)/N(c)$. Since $N(c)$ is unable to be measured, here we estimate a reliable upper bound on this number, which is $N_{max}(c)$, a maximum number of biomolecules on

average that can be adsorbed on the sensor surface. Note that the sensitivity, $\Delta I(c)/ N_{max}(c)$ will be lower bound on the true sensitivity $\Delta I(c)/N(c)$ because $N(c) \leq N_{max}(c)$. First, we derive $N_{max}(c)$ by considering the illuminated beam area on the sensor surface. The illuminated beam diameter is around 2 μm, thus the effective sensor area is 4 μm^2. It shows that only a small fraction of the total population of cTnI molecules will end up adsorbed on the illuminated sensor area. We will assume that the adsorbed molecules are equally distributed across the entire sensor surface, which has a dimension of 3 mm x 3 mm, and an area of 9 mm^2.

Hence, given a certain maximum possible adsorbed population on the surface, only a fraction of 4 $\mu m^2/9 \times 10^6\ \mu m^2 = 0.444 \times 10^{-6}$ will be in the sensing area and relevant to the Raman intensity shift. Initially, there are c (9 mm^3 /1L) x 6.022 × $10^{23}\ M^{-1} = 5.42\ c \times 10^{18}\ M^{-1}$ biomolecules on the sensor. If all the molecules are to be adsorbed on the sensor surface, on average 0.444×10^{-6} of the total would be in the sensor area. Therefore, $N_{max}(c) = 24\ c \times 10^{13}\ M^{-1}$

In Fig. 4e, we plotted N_{max} versus $\Delta I(c)$ for the measured values of c from 380 fM to 378 pM, corresponding to N_{max} ranging from 91 to 90720 molecules. The relationship between N_{max} and $\Delta I(c)$ is nonlinear, which is consistent with the following fitting function (red curve),

$$N_{max} = A_1\left(e^{\Delta I/I_1} - 1\right) + A_2\left(e^{\Delta I/I_2} - 1\right) \qquad (1)$$

where A_i, I_i, i=1, 2, are the fitting parameters. The best fitting values are A_1=2, A_2=9 x 10^2, I_1=350, and I_2=900. Since there are clearly two exponential regimes in the data, a biexponential fitting function is required. The fitting function is not just a sum of two exponentials but includes a constant term - (A1+A2) so that N_{max}=0 when ΔI = 0. The observed nonlinearity is due to the possibility of multiple adsorbed molecules on the sensor leading to interference effects (when c

increases) and hence, with each additional molecule having a decreasing impact on the Raman intensity shift. The obtained sensitivity ($\Delta I(c)/N_{max}(c)$) is 0.692 for 380 fM and this value decreases with increasing c due to nonlinearity. According to this analysis, it can be concluded that the sensor operates in the few molecules (<100) detection regime.

Supplementary Note 4. Enhancement Factor calculations

Since the proposed TPP cavity surface consists of random nanostructures, we calculate the spatially averaged Enhancement Factor (***EF***) using a well-established method[4-7]. In our experiment, we compared 100 µM cyanine 7.5 (cy7.5) based SERS intensity with a thin liquid layer of cy7.5.

$$EF = (I_{SERS}/N_{SERS})/(I_{Bulk}/N_{Bulk}) \quad (2)$$

In Eq. (2), I_{bulk} and I_{SERS} represent the intensity values at the scattering band of interest (e.g., 943 cm^{-1}) in the bulk liquid Raman spectrum and SERS spectrum, respectively. N_{Bulk} corresponds to the number of cy7.5 molecules in the bulk solution contributing to the unenhanced Raman signal, while N_{SERS} refers to the number of chemisorbed cy7.5 molecules on the substrate contributing to the SERS signal. The values of N_{Raman} and N_{Bulk} can be determined using the following equations:

$$N_{SERS} = A_{beam} * R * \mu \quad (3)$$

$$N_{Bulk} = A_{beam} * H * \rho \quad (4)$$

where A_{beam} represents the area of the laser beam, R is the fractional surface area (ratio of exposed surface area to the nanostructure unit cell), μ is the packing density of cy7.5 molecules on the

surface of the substrate, H is the apparent height of the cy7.5 liquid layer emitting the Raman signal, and ρ is the molecular density of the prepared cy7.5 solution.

EF can be written as:

$$EF = (H * \rho / (R * \mu)) * (I_{SERS} / I_{Bulk}) \quad (5)$$

In our case, the estimated values of parameters used in Eq. (5) are μ = 6.261 x 10^{18} molecules/cm^2, R = 0.325, H =16 x 10^{-4} cm, and ρ = 6.023 x 10^{21} molecules/cm^3, I_{SERS} (normalized to laser power and concentration) = 16779 x 10^5 counts, I_{Bulk} = 226 counts. The estimated enhancement factor of the TPP substrate is ~3.52 x 10^7.

Supplementary Note 5. ELISA test for benchmarking

We have conducted an ELISA test for benchmarking the performance of the proposed TPP cavity-based SERS substrate. In Supplementary Table 1, Column 1 represents the actual known concentration of cTnI protein spiked in plasma. Column 2 is its corresponding measured ELISA absorbance. The final concentration of cTnI protein (shown in column 3) was detected by ELISA after interpolating from the calibration plot, as shown in Supplementary Fig. 19. Due to the limitation of the ELISA kit, we could detect in the range of ~166 pM to 2.6 pM. Accordingly, higher concentrations of spiked cTnI samples were prepared and studied followed by calibration and normalization to obtain the calculated spiked cTnI concentration detected by ELISA. The ELISA results were comparable for higher concentrations (picomolar), but the ELISA kit was not able to provide any results for lower concentrations (femtomolar).

Supplementary Fig. 19 shows the calibration plot of ELISA with interpolated spiked cTnI samples (in red). As can be seen, the sensitivity of the TPP-SERS platform for protein detection is comparable with the gold standard, ELISA method for higher concentration. Note that ELISA could only detect up to 2.6 pM, which is a much higher cTnI protein concentration than what was detected using TPP substrate, which is in the femtomolar range. In addition, ELISA measurement takes a much longer time and involves multiple washing steps. SERS methods provide a relatively faster means for sensitive detection.

Supplementary Note 6. SERRS with nanopatterned TPP cavities

We also conducted SERRS experiments using nanopatterned TPP cavities. Two-dimensional (2D) periodic nanohole grating with a hole diameter of 80 nm and different grating periods (200 nm to 350 nm) were fabricated on the top Au layer (grating area=30 μm x 30 μm). A SEM image of the fabricated 2D grating with period 250 nm is shown in the inset of Supplementary Fig. 21a. To obtain a relatively smooth Au surface on the Sb_2S_3 layer with a good aspect ratio Au grating, the thickness of the Au layer was chosen as 30 nm. The nanopatterned TPP cavities were annealed at 210°C to realize second-order TPP resonance around 785 nm for SERRS. As shown in Supplementary Fig. 21a, TPP modes are excited in both bandgaps of DBR, and the resonance wavelength of both TPP modes can be tuned by changing the period of the grating, since the effective index of nanograting varies with change in the grating period. It is worth mentioning that TPP mode can also be excited within the bandgap of DBR by nanopatterning[8].

In Supplementary Fig. 21b, we show the SERRS spectrum of scalable and nanopatterned TPP cavities using 10 μM cy7.5 dye concentration. In contrast to the 10 nm Au film-coated TPP cavity (Fig. 4b), a lower Raman signal is obtained for the 30 nm Au film-coated TPP cavity due

to decreased surface roughness, which in turn reduces the field enhancement and hence the Raman signal enhancement. Nevertheless, nanopatterned TPP cavity shows large Raman signal enhancement compared to scalable TPP cavity due to nanopatterning, as a periodic nanohole array is an excellent substrate for SERS applications because a huge amount of electromagnetic energy can be localized in nanoholes due to repeated reflections of the trapped incident light[9]. We simulated the electric field intensity distribution of nanopatterned TPP cavity at TPP resonance wavelength and observed similar characteristics (see Supplementary Fig. 22).

References

1. Dubey, A. et al. Demonstration of a superior deep-UV surface-enhanced resonance Raman scattering (SERRS) substrate and single-base mutation detection in oligonucleotides. *J. Am. Chem. Soc.* **143**, 19282-19286 (2021)
2. Jha, S. K. et al. Deep-UV surface-enhanced resonance Raman scattering of adenine on aluminum nanoparticle arrays. *J. Am. Chem. Soc.* **134**, 1966−1969 (2012)
3. Smith, W. E. & Rodger, C. Surface Enhanced Raman Scattering (SERS), Applications, Encyclopedia of Spectroscopy and Spectrometry (Second Edition), (Academic Press 1999), Pages 2822-2827
4. Cai, W. B., et al. Investigation of surface-enhanced Raman scattering from platinum electrodes using a confocal Raman microscope: dependence of surface roughening pretreatment. *Surf Sci.* **406**, 9-22 (1998)
5. Smythe, E. J., Dickey, M. D., Bao, J., Whitesides, G. M., Capasso, F. Optical Antenna Arrays on a Fiber Facet for in Situ Surface-Enhanced Raman Scattering Detection *Nano Lett*. **9** 1132-1138 (2009)
6. Dinish, U. S., Yaw, F. C., Agarwal, A. & Olivo, M. Development of highly reproducible nanogap SERS substrates: comparative performance analysis and its application for glucose sensing. *Biosens. Bioelectron.* **26**, 1987 (2011)
7. Perumal J., et al. Design and fabrication of random silver films as substrate for SERS based nano-stress sensing of proteins. *RSC Adv*. **4**, 12995 (2014)

8. Buchnev, O., Belosludtsev, A. Reshetnyak, V., Evans, D. R & Fedotov, V. A. Observing and controlling a Tamm plasmon at the interface with a metasurface. *Nanophotonics* **9,** 897–903 (2020)
9. Zeng, Y. et al. Light-trapped nanocavities for ultraviolet surface-enhanced Raman scattering. *The Journal of Physical Chemistry C* **125**, 17241-17247 (2021)
